# Analyses of Copy Number Variation of GK Rat Reveal New Putative Type 2 Diabetes Susceptibility Loci

**DOI:** 10.1371/journal.pone.0014077

**Published:** 2010-11-23

**Authors:** Zhi-Qiang Ye, Shen Niu, Yang Yu, Hui Yu, Bao-Hong Liu, Rong-Xia Li, Hua-Sheng Xiao, Rong Zeng, Yi-Xue Li, Jia-Rui Wu, Yuan-Yuan Li

**Affiliations:** 1 Key Laboratory of Systems Biology, Shanghai Institutes for Biological Sciences, Chinese Academy of Sciences, Shanghai, China; 2 Shanghai Center for Bioinformation Technology, Shanghai, China; University of Leuven, Belgium

## Abstract

Large efforts have been taken to search for genes responsible for type 2 diabetes (T2D), but have resulted in only about 20 in humans due to its complexity and heterogeneity. The GK rat, a spontanous T2D model, offers us a superior opportunity to search for more diabetic genes. Utilizing array comparative genome hybridization (aCGH) technology, we identifed 137 non-redundant copy number variation (CNV) regions from the GK rats when using normal Wistar rats as control. These CNV regions (CNVRs) covered approximately 36 Mb nucleotides, accounting for about 1% of the whole genome. By integrating information from gene annotations and disease knowledge, we investigated the CNVRs comprehensively for mining new T2D genes. As a result, we prioritized 16 putative protein-coding genes and two microRNA genes (*rno-mir-30b* and *rno-mir-30d*) as good candidates. The catalogue of CNVRs between GK and Wistar rats identified in this work served as a repository for mining genes that might play roles in the pathogenesis of T2D. Moreover, our efforts in utilizing bioinformatics methods to prioritize good candidate genes provided a more specific set of putative candidates. These findings would contribute to the research into the genetic basis of T2D, and thus shed light on its pathogenesis.

## Introduction

Type 2 diabetes (T2D), formally known as non-insulin-dependent diabetes, accounts for about 90% of the 180 million diabetic cases around the world [1]. Characterized by defects in both insulin secretion from pancreatic islet beta-cells and insulin action in peripheral tissues, this chronic and complex disorder is supposed to be predisposed by the combined action of multiple genetic factors [2], [3]. In the last two decades, large efforts including traditional candidate gene mapping and recent high-throughput genome-wide association studies were performed to unveil the genetic basis of T2D, and have found nearly 20 human T2D genes [3]–[8] and a number of related loci in human, mouse and rat genomes [9], [10]. However, the precise molecular pathogenesis of this heterogeneous disease remains poorly characterized, and more T2D-related genes are expected to be uncovered.

The Goto-Kakizaki (GK) rat, a nonobese animal model of T2D, was developed by repeated inbreeding of glucose-intolerant Wistar rats [11]. During their development, GK rats suffer from reduced beta-cell mass and insulin resistance spontaneously, and thus provide a feasible opportunity to search for susceptible loci, investigate pathogenesis and develop therapeutic strategies [12], [13]. Several quantitative trait locus (QTL) analyses on this model have already identified a number of genomic loci harboring susceptible variants [14]–[16].

While most disease-association studies of genetic variation focused on individual nucleotide sequences, large-scale changes like copy number variations (CNVs), generally defined as the copy number differences of DNA stretches larger than 1 Kb, have also been linked to dozens of human diseases [17]. Among the most well-known cases is the association of Down Syndrome with an extra copy of chromosome 21 identified by karyotype technology [18]. New high-throughput approaches like array-based comparative genome hybridization (array CGH, or aCGH) have allowed the identification of CNVs in the whole genome [19]–[21], and have discovered that CNVs are extensively distributed along the chromosomes. Some of the CNVs are found to be implicated in complex diseases including neuropsychiatric, autoimmune diseases and so on [22]–[25], but the association of CNVs with T2D remains largely unexploited except that a very recent study confirmed the implication of a previously identified human gene *TSPAN8* [26].

In this work, we conducted a genome-wide screen for CNVs between GK (T2D model) and Wistar rat (wild type) using array CGH. A non-redundant set of CNV regions with the total length of about 36 Mb was identified, including several novel T2D susceptibility loci involving 16 protein-coding genes (*Il18r1*, *Cyp4a3*, *Sult2a1*, *Sult2a2*, *Sult2al1*, *Nos2*, *Pstpip1*, *Ugt2b*, *Uxs1*, *RT1-A1*, *RT1-A3*, *RT1-Db1*, *RT1-N1*, *RT1-N3*, *RT1-O*, and *RT1-S2*) and two microRNA genes (*rno-mir-30b* and *rno-mir-30d*). It is so far the first investigation of T2D in GK rats from the viewpoint of copy number variation on a genome-wide scale, and the CNVs identified in GK rats are supposed to shed light on the genetic basis and pathogenesis of T2D.

## Results

### Array Data Processing

The comparison between GK and Wistar rats had three biological replicates (“forward”) with a dye swap (“reverse”) for each, resulting in 6 arrays. The array data were processed following the procedures described in Materials and Methods. In the step of quality control, a set of data from a small region on the sixth chip involving 1278 spots (∼0.5%), in addition to 152, 157, 147, 143, 155, and 401 scattered spots of each chip respectively (∼0.08% on average), were filtered out due to poor qualities. To detect the bias of dye labeling, we hierarchically clustered all 6 chips based on the logarithm ratios (M values). It turned out that all “forward” chips were distinctly separated from “reverse” ones, conflicting to the expectation that a dye-swap pair ought to be grouped together (Figure S1A). Using a linear model, we found that about 23.7% of the spots showed significant dye bias (*p*<0.05). After the correction of dye bias, the new clustering result agreed with the expectation (Figure S1B). Three dye-swap pairs resulted in three sets of M values, each of which corresponded to one GK replicate. The chromosomes were then partitioned according to the smoothed M values of the probes tiled on them, a process formally termed as segmentation. The raw intensities and processed data have been deposited in NCBI's Gene Expression Omnibus [27] and are accessible through GEO Series accession number GSE21387.

### CNVR Identification

According to our definition of CNV regions (CNVRs, see Materials and Methods), we identified about 101 CNVRs in each GK rat on average, covering approximately 26 M base pairs, *i.e.*, about 1% of the rat genome (Table S1 and S2), whose order of magnitude was the same as the overall length of published rat CNVRs (22 Mb and 15.5 Mb on two different platforms) [21]. The comparison of the CNVRs identified from the 3 samples turned out that the majority of them (greater than 70%) were consistent among individuals (Table S2 and S3). We then merged the CNVRs from all the 3 samples to a final non-redundant set, comprising 137 CNVRs, covering 36.31 Mb (Table S4 and the “Non-redundant” column of Table S2). Follow-up investigations were based on this non-redundant data set.

**Figure 1:**
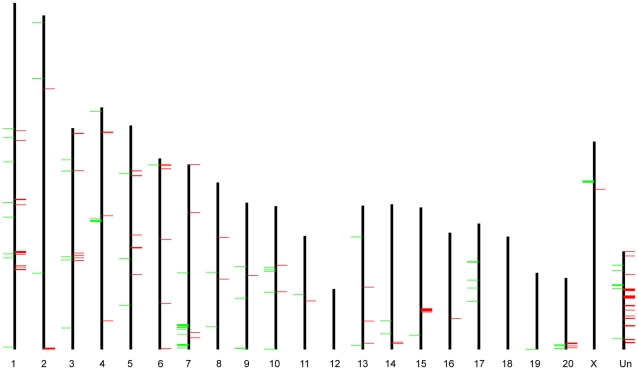
Chromosomal distribution of GK/Wistar CNVRs. Green bars on the left and red bars on the right of chromosomal axes represent CNV “loss” and “gain”, respectively. Chromosome “Un” represents the pseudo-chromosome consisting of contigs that can not be confidently mapped to a specific chromosome.

We plotted the GK/Wistar CNVRs along each chromosome (Figure 1), and found that they were non-uniformly distributed with the extreme cases that chromosome 12 and 18 contained none, while chromosome 7 and 15 contained more CNVRs than random (4.5 Mb and 2.7 Mb identified *vs.* only 1.8 Mb and 1.4 Mb expected, respectively). The non-uniform pattern of CNVRs' distribution was similar to some extent with the previous report of rat CNVRs [21].

### Investigation into CNVR

In order to mine the genetic variations underpinning the phenotypic difference between GK and Wistar rats (*i.e.*, diabetic and non-diabetic), we investigated the CNVRs through examining their overlapping with various genomic features such as protein-coding genes and microRNAs. Genomic features covered by the GK/Wistar CNVRs served as a valuable repository for exploring genetic factors that play roles in pathogenesis of T2D through altered copy numbers and thus abnormal expression levels. For a CNV gene already reported to be T2D-related, our work could help elucidate its underlying mechanisms, *i.e.*, the gene dosage effect via copy number variation. More importantly, CNV genes that were not known to be related to T2D might contain novel candidates, and it was supposed to be promising to sort out them from unrelated ones by combining prior biological knowledge using bioinformatics methods.

### Gene and Intergenic Content of CNVRs

Gene and intergenic regions in the GK/Wistar CNVRs were determined according to the chromosomal locations of rat genes obtained from NCBI Entrez Gene. Taken together, regions of 3.22 Mb were annotated by Entrez Gene, accounting for only 8.87% of all CNVRs, much less than the proportion of gene regions in the whole genome (21.35%, Table 1). It seemed that copy number variation would preferentially reside in intergenic regions. We further carried out a random simulation to test the statistical significance (see Materials and Methods), and found that the overlapping magnitude between randomized intervals and genes was significantly larger than that between real CNVRs and genes (7.84 Mb expected in random with a standard deviation of 1.13 Mb *vs.* 3.22 Mb in fact, *p*<1e-10), which supported our inference about the preference of CNVRs to intergenic regions. This could be partly explained by the purifying selection, which probably acted on the GK/Wistar CNVs during the process of selective breeding. Variations in most gene regions might be more likely to have deleterious effects than those in intergenic regions, and the stronger negative selection pressure on gene regions might thus result in the lower observed frequency of CNVs in the gene regions than in the intergenic regions.

**Table 1:** Gene and intergenic constitution in CNVRs and whole genome.

	Status	All	Gene Region	Intergenic Region
CNVR (Mb)	Gain	22.75 (100%)	1.91 (8.40%)	20.84 (91.60%)
	Loss	13.56 (100%)	1.31 (9.66%)	12.25 (90.34%)
	Total	36.31 (100%)	3.22 (8.87%)	33.09 (91.13%)
Genome (Gb)		2.83 (100%)	0.60 (21.35%)	2.23 (78.65%)

### Functional Analysis of CNV Genes

A total of 62 and 72 Entrez genes were identified in “gain” and “loss” CNVRs respectively (Table S4 and S5). We checked whether these genes contained any of those previously reported to be related to T2D. A list of 425 known T2D-related genes from published sources was collected (see Materials and Methods) and compared with these 134 GK/Wistar CNV genes, but none in common was found. This result indicated that the potential CNV factors behind the diabetic pathogenesis of GK rats might be other genes whose relationships with T2D had not been observed. They could be genes that had been annotated in NCBI Entrez Gene or even loci that had never been identified before. In this work, we focused on mining candidates from known genes (i.e., 134 CNV genes) according to the aforementioned strategy of adopting prior knowledge.

We first utilized the knowledgebase of KEGG pathways so as to identify potential novel candidates that are supposed to be related to the characteristics of diabetes. After mapping the 134 CNV genes to KEGG, we found 41 pathways were associated with those genes (Table S6). The CNV genes falling in the T2D-related pathways were then selected for further analysis (Table 2). Since T1D shares some characteristics with T2D, and the involvement of sulfur metabolism in diabetes has been reported previously [28], [29], the CNV genes (*RT1-A1*, *RT1-A3*, *RT1-Db1*, *RT1-N1*, *RT1-N3*, *RT1-O*, *RT1-S2*, *Sult2al1*, *Sult2a1*, and *Sult2a2*) in pathways of “type I diabetes mellitus (04940)” and “sulfur metabolism (00920)” were prioritized. Sugar or fatty acid metabolisms and PPAR signaling pathways are well known to be T2D-relevant [2], and thus the CNV genes (*Uxs1*, *Ugt2b*, and *Cyp4a3*) in pathways of “starch and sucrose metabolism (00500)”, “pentose and glucuronate interconversions (00040)”, “fatty acid metabolism (00071)”, and “PPAR signaling (03320)” were preferentially selected as well. We also found that *Ugt2b*, *Cyp4a3*, and sulfur metabolism-related CNV genes (*Sult2a1*, *Sult2a2*, and *Sult2al1*) overlapped the T2D QTLs of Niddm37, Niddm25 and Niddm44, respectively, strengthening the hypothesis that these genes may confer susceptibility to T2D.

**Table 2:** Selected GK/Wistar CNV genes involved in diabetes-related pathways.

KEGG pathway (ID)	Status	CNV Gene
Type I diabetes mellitus (04940)	Gain	*RT1-A1*, *RT1-A3*, *RT1-Db1*
	Loss	*RT1-N1*, *RT1-N3*, *RT1-O*, *RT1-S2*
Sulfur metabolism (00920)	Gain	*Sult2al1*, *Sult2a1*, *Sult2a2*
Starch and sucrose metabolism (00500)	Gain	*Uxs1*
	Loss	*Ugt2b*
Pentose and glucuronate interconversions (00040)	Loss	*Ugt2b*
Fatty acid metabolism (00071)	Loss	*Cyp4a3*
PPAR signaling pathway (03320)	Loss	*Cyp4a3*

We noticed that the GK/Wistar CNV genes were significantly overrepresented in the pathways of olfactory transduction (04740), immune response (05332, 05330, 05320, and 04612), and cell adhesion molecule (04514) (Table S6). They were reminiscent of the functional enrichment results previously reported in CNV genes of human, mouse and rat, indicating that the bias towards these functional categories might be a common overall characteristic of CNV genes [19], [21], [30].

Since there are complex relationships between diseases, constituting a “diseasome” [31], diseases directly related to T2D (“near-T2D”) could help understand the relatedness of GK/Wistar CNV genes to T2D. A total of 1097 “near-T2D” genes were retrieved following the procedure described in Materials and Methods. Due to the complexity and heterogeneity of diabetes, we speculated that a part of these genes might also contribute to some extent to T2D. In fact, five “near-T2D” genes including *Pstpip1*, *Il18r1*, *Sult2a1*, *Nos2*, and *RT1-Db1*, occurred in the GK/Wistar CNV gene list (Table S7). We checked the relationship between them and rat QTLs as well, and found that they overlapped QTLs of “blood pressure”, “serum triglyceride”, “serum cholesterol”, or “body weight”. Specifically, *Pstpip1*, *Il18r1*, *Sult2a1* overlapped the QTLs of “Non-insulin dependent diabetes mellitus (Niddm)”.

There are several web tools for prioritizing human disease candidate genes from given genomic intervals [32]. The comparison between our 134 rat CNV genes and the 103 human T2D genes prioritized by Tiffin *et al.* [33] found one common gene, *Uxs1*, which was also sorted out by our T2D-relevant pathway mapping.

Taken together, the above analysis highlighted 16 GK/Wistar CNV genes, providing a valuable collection of most likely disease candidates to be prioritized for further experiments (Table S8). These genes are unequally distributed on several different chromosomes and different CNV regions. Among them, all the 7 *RT1* genes are clustered in two regions of chromosome 20, *i.e.*, *RT1-A1*, *RT1-A3*, and *RT1-Db1* in one cluster (the gap between cnv.gain.54 and cnv.gain.53 is only ∼345 Kb according to Table S4) while *RT1-N1*, *RT1-N3*, *RT1-O*, and *RT1-S2* in the other one. However, the 3 genes in the first cluster are only supported by one sample, which reduced our confidence that these genes are likely to contribute to susceptibility to T2D. The three *Sult2a* genes are clustered in a gain region on chromosome 1, and are supported by all three samples. The other six genes are distributed on six different chromosomes respectively, with *Uxs1*, *Cyp4a3*, and *Nos2* identified in all three samples. These data will offer additional clues for the follow-up prioritization of these 16 genes.

### Ultraconserved Elements and MicroRNAs

According to the aforementioned analysis, more than 90% of the CNVRs were intergenic regions. Although the intergenic regions, as well as non-coding gene regions such as introns and UTRs, may not involve as many functional features as coding regions, recent studies demonstrated that some of non-coding regions can play important regulatory roles. We therefore extended our focus beyond coding regions to include ultraconserved elements and microRNAs.

Ultraconserved elements are defined as stretches of DNA (longer than 200 bp) which are extremely conserved in orthologous regions of the human, rat and mouse genomes. It has been speculated that they are under strong purifying selection, and may play important roles in DNA binding, RNA processing and transcriptional regulation [34]. We compared the GK/Wistar CNVRs and 481 published ultraconserved elements (126.7 Kb in total mapped to rat genome assembly rn4), but no overlap was found, while ∼1.5 Kb would be expected by chance (*p* = 0.027), consistent with the conclusions reported in other studies [21], [35]. These results in combination with the above gene and intergenic content of CNVRs, supported the opinion that, as a global trend, most GK/Wistar CNVs were found in non-functional rather than functional regions, probably due to the purifying selection during the selective breeding.

**Figure 2:**
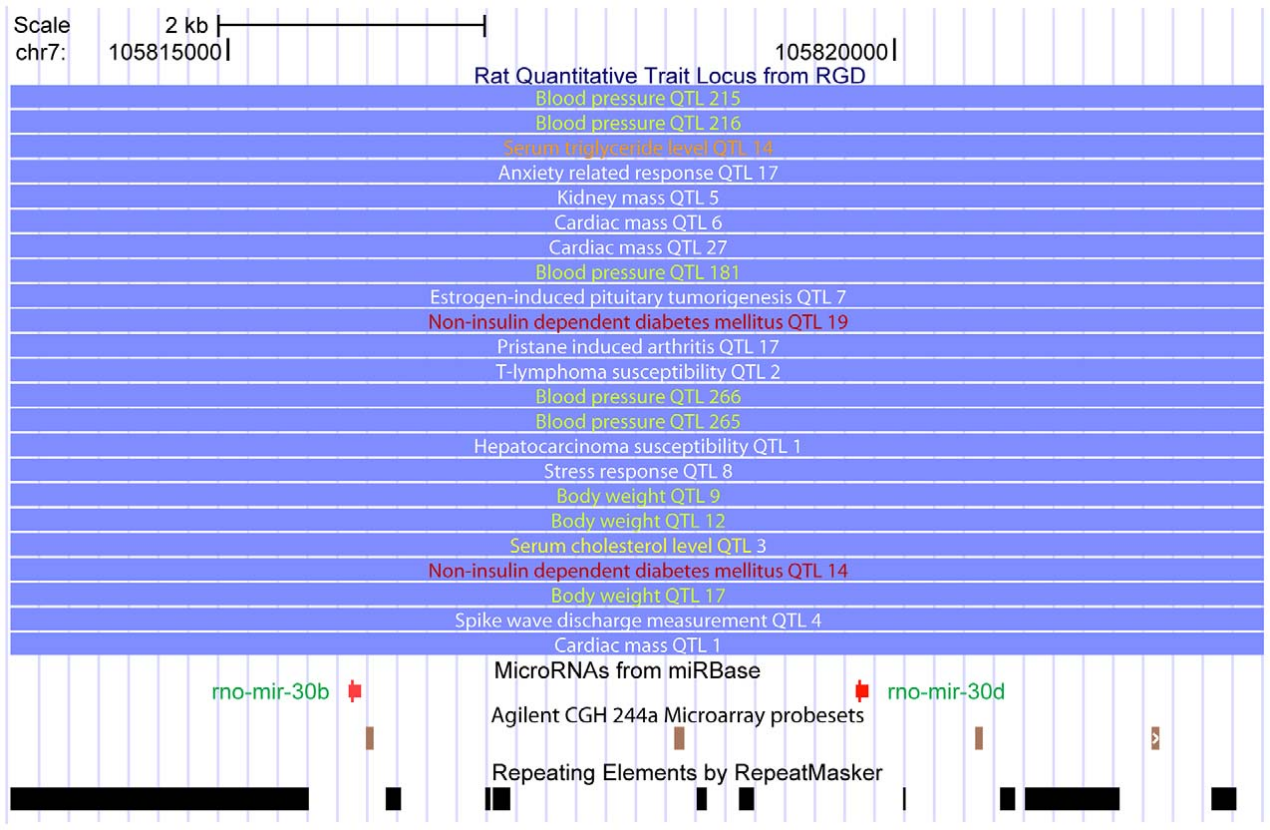
The microRNA *rno-mir-30b* and *rno-mir-30d* located in T2D QTLs. The QTLs of Niddm (Non-insulin dependent diabetes mellitus) 14 and 19 cover these 2 microRNAs. In addition, there are many other QTLs like “serum triglyceride 14”, “serum cholesterol 3”, “blood pressure 181/215/216/265/266”, “body weight 9/12/17” in this region, and these traits are known to be related to diabetes. This figure was prepared using UCSC genome browser.

Considering the significant contributions of microRNAs to development processes and the pathogenesis of diseases at the post-transcriptional level [36]–[37], we examined if there were any microRNA genes in our GK/Wistar CNVRs. By comparing the genomic positions of known rat microRNA genes with those of GK/Wistar CNVRs, we found that *rno-mir-30b* and *rno-mir-30d* were simultaneously covered by a “gain” region on chromosome 7 in all three samples (Table S9) within a region of only 3.8 Kb. Interestingly, several T2D QTLs and various relevant QTLs including body weight, blood pressure, and serum triglyceride level QTLs were also located in this region, implying that copy number variation of these microRNA genes could be correlated to these quantitative traits (Figure 2). A recent publication reported that altered expression of *mir-30d*, as a response to glucose, influences insulin gene expression in mouse Min6, a pancreatic island cell line [38]. Although further investigations are still needed, we obtained additional evidence supporting the involvement of *mir-30b/30d* in T2D pathogenesis by means of copy number variation.

To further elucidate the putative roles of *mir-30b/30d*, we looked at their predicted targets using MicroCosm [39]. Taken together, there were 1868 and 1776 targets for *rno-mir-30b* and *rno-mir-30d*, respectively. Like the analysis performed on CNV genes, we compared these target genes with the 425 T2D-related genes. It turned out that 39 and 35 targets of *mir-30b* and *mir-30d* occurred in this T2D gene list respectively, and were both significantly overrepresented (*p* = 0.000273 and 0.00152, detailed targets listed in Table 3), supporting the hypothesis of *mir-30b* and *mir-30d*'s involvement in T2D. Among them, *Pparg* and *Akt2* (targets of *mir-30b*), *Hnf1b*, *Hnf4a*, and *Lmna* (targets of *mir-30d*), are well-known genes implicated in T2D or insulin resistance. We then mapped these microRNA targets to KEGG pathways, and found that 5(2), 12(6), 10(4), 14(12), 4(5) and 1(3) targets of *mir-30b*(*mir-30d*) belonged to the pathways of “type II diabetes (04930)”, “Type I diabetes (04940)”, “pancreatic cancer (05212)”, “insulin signaling (04910)”, “PPAR signaling (03320)” and “maturity onset diabetes of the young (04950)”, respectively (Table 4). Moreover, several fatty acid or sugar-related metabolism pathways (00010, 00030, 00512, 00051, 00071, and 01030) were enriched with these predicted targets with considerably low *p* values (Table S10 and Table S11). These results provided extra evidence of a role for *mir-30b/30d* in diabetes pathogenesis.

**Table 3:** Targets of *rno-mir-30b* and *rno-mir-30d* in T2D-related genes.

microRNA	Predicted targets
*rno-mir-30b*	*Aire*, *Akt2* *, *Bud13*, *Cblb*, *Cdc123*, *Eif4e*, *Elf1*, *Fgb*, *Gcg*, *Hdac3*, *Irf4*, *Kcnj5*, *Klrg1*, *Mapk8*, *Med14*, *Mgea5*, *Mttp*, *Neurod1*, *Nfkb1*, *Nmu*, *Parl*, *Pbx1*, *Pfkl*, *Pik3r2*, *Pparg* *, *Ppargc1b*, *Prkce*, *Prmt2*, *Rapgef4*, *Rpa2*, *Rrad*, *Serpine1*, *Slc2a10*, *Socs1*, *Srebf1*, *Tlr4*, *Ubl5*, *Ucp2*, *Wdr42a*
*rno-mir-30d*	*Ace*, *Cblb*, *Cdh15*, *Cp*, *Cyb5r4*, *Egfr*, *Foxo1*, *Hdac3*, *Hnf1b* *, *Hnf4a* *, *Inpp5k*, *Irf4*, *Lgr5*, *Lmna* *, *Neurod1*, *Nfkb2*, *Nfkbia*, *Nr1i3*, *Nr4a1*, *Parl*, *Pbx1*, *Pik3r2*, *Ppargc1b*, *Ppp1r3d*, *Prkar2b*, *Ptf1a*, *Rbp4*, *Rrad*, *Sell*, *Sirt1*, *Slc2a10*, *Socs1*, *Sorcs1*, *Srebf1*, *Tlr4*

*Well-known genes implicated in T2D or insulin resistance.

**Table 4:** The targets of *rno-mir-30b* and *rno-mir-30d* involved in diabetes-related pathways.

KEGG pathway	microRNA	Targets
* Glycolysis/Gluconeogenesis	*rno-mir-30b*	*Aldoc*, *Gapdh*, *Ldhb*, *Pgm1*, *Aldh3a1*, *Pfkl*, *Aldh1a3*, *LOC291543*, *Gpi*, *LOC294844*, *Aldh2*, *RGD1561178*, *Adh4*, *Aldh1a7*, *LOC303448*, *RGD1563446*, *LOC366864*, *RGD1566272*, *RGD1564688*, *RGD1565928*, *RGD1562758*, *RGD1559704*, *LOC499896*, *LOC500912*, *RGD1565368*, *LOC680538*, *LOC682005*, *LOC685186*, *LOC688677*, *Gapdh-ps2*
	*rno-mir-30d*	*Fbp2*, *Adh7*, *Adh1*, *Ldhb*, *Pgk1*, *Pgm1*, *Aldh3a1*, *LOC291543*, *RGD1561881*, *Aldh2*, *Adh4*, *RGD1565238*
* Pentose phosphate pathway	*rno-mir-30b*	*Aldoc*, *Pgm1*, *Pfkl*, *Gpi*, *Tkt*
O-Glycan biosynthesis	*rno-mir-30b*	*Ogt*, *Galnt13*, *Galnt3*, *Galnt1*
	*rno-mir-30d*	*Ogt*, *Galnt13*, *Galnt3*, *C1galt1*
Fructose and mannose metabolism	*rno-mir-30b*	*Aldoc*, *Pfkfb1*, *Pfkl*, *Mpi*, *Fpgt*
Fatty acid metabolism	*rno-mir-30d*	*Adh7*, *Adh1*, *Cpt2*, *Aldh2*, *Adh4*, *Dci*, *Acox3*
Glycan structures - biosynthesis 1	*rno-mir-30d*	*Ogt*, *Man1a1*, *Chst1*, *Ext1*, *B4galt4*, *Galnt13*, *Ddost*, *Galnt3*, *C1galt1*, *Mgat5*, *Hs3st1*, *Chst3*, *Mgat2*
Insulin signaling pathway	*rno-mir-30b*	*Braf*, *Mapk8*, *Eif4e*, *Cblb*, *Pygm*, *Akt2*, *Socs1*, *Pfkl*, *Pik3r2*, *Calml3*, *Cep152*, *Rps6kb2*, *Srebf1*, *Pik3cb*
	*rno-mir-30d*	*Fbp2*, *Cblb*, *Calm1*, *Calm3*, *Prkar2b*, *Pygm*, *Socs1*, *Pik3r2*, *Phkb*, *Calm2*, *Srebf1*, *Foxo1*
PPAR signaling pathway	*rno-mir-30b*	*Pparg*, *Slc27a6*, *Gk*, *Cyp8b1*
	*rno-mir-30d*	*Cpt2*, *Slc27a6*, *Gk*, *Fabp4*, *Acox3*
Type II diabetes mellitus	*rno-mir-30b*	*Mapk8*, *Socs1*, *Prkce*, *Pik3r2*, *Pik3cb*
	*rno-mir-30d*	*Socs1*, *Pik3r2*
Pancreatic cancer	*rno-mir-30b*	*Braf*, *Mapk8*, *Erbb2*, *Akt2*, *Pik3r2*, *Brca2*, *Rac2*, *Rad51*, *Nfkb1*, *Pik3cb*
	*rno-mir-30d*	*Egfr*, *Pik3r2*, *Brca2*, *Rad51*
Maturity onset diabetes of the young (MODY)	*rno-mir-30b*	*Neurod1*
	*rno-mir-30d*	*Hnf1b*, *Hnf4a*, *Neurod1*
Type I diabetes mellitus	*rno-mir-30b*	*RT1-A1*, *RT1-A2*, *RT1-Cl*, *RT1-CE12*, *RT1-CE1*, *RT1-A3*, *RT1-M6-2*, *RT1-CE14*, *RT1-CE4*, *RT1-M6-1*, *RT1-CE16*, *Ica1*
	*rno-mir-30d*	*Gzmb*, *Il1a*, *Ifng*, *RT1-Db1*, *H2-Ob*, *Cd86*

*Significantly or nearly significantly enriched, *p*<0.10.

## Discussion

In this study, we identified a catalogue of CNVRs between GK and Wistar rats using tiling array CGH. Given the hypothesis that the phenotypic difference between GK and Wistar (diabetic and non-diabetic) ought to be attributed in a large part to their genomic variations, we carried out a series of bioinformatics functional analysis on these GK/Wistar CNVRs to narrow down the scope for further exploration of T2D candidate genes. A total of 16 protein-coding genes and 2 microRNA genes were prioritized for further analysis (Table S8 and Table 3), which might in combination or alone contribute to the pathogenesis of diabetes on the basis of varied copy number in the genomic level.

We believe that the list of GK/Wistar CNVRs is a valuable repository for mining genetic factors that play roles in pathogenesis of T2D through altered copy numbers. In the bioinformatics analysis, we focused on the Entrez protein-coding genes and microRNAs with known genomic locations, and found 134 protein-coding genes and 2 microRNAs implicated in GK/Wistar CNVRs. By integrating available knowledge about T2D, we prioritized 16 protein-coding genes and 2 microRNAs as good candidates for further experiments for validating their contribution to the pathogenesis of T2D by means of dosage effect. In addition, CNVRs without annotations of Entrez genes or microRNAs may also be worth further investigation. We checked other gene annotations from UCSC “KnownGene”, “RefGene”, “mRNA”, “EST”, and “EnsemblGene”, and found various coding signals outside the scope of Entrez gene annotations (Table S12). They could be novel genes, but additional evidence is needed.

Among the preferentially selected protein-coding genes, *Il18r1* was previously identified as a T2D candidate gene in a cohort of African American families [40]. The gene *Cyp4a3* is involved in fatty acid metabolism and Pparg signaling, disorders of which are closely related to the pathogenesis of T2D [2], [41]. As mentioned in the results, several sulfur-containing compounds are used in the therapy of diabetes while the therapeutic mechanism is not yet clear. Thus the identification of *sult2a1*, *sult2a2* and *sult2al1* echoes the beneficial effects of sulfur-containing compounds, and further study might provide insights into the mechanism.

We analyzed a public dataset GSE13271 [42], currently the only GK/Wistar differential expression dataset in NCBI GEO, and identified 30 sets of differentially expressed genes (DEGs) between GK and Wistar rats corresponding to different tissues, time points, and feeding conditions. When comparing these DEGs with the GK/Wistar CNV genes identified in this work, we found that some of them showed consistency in certain tissues (Table S13), including *RT1-N1* in muscle, *Ugt2b* in liver, *RT1-A3* in all three tissues (liver, fat, and muscle). There was also inconsistency between CNV and differential expression, including *Sult2al1*, *Sult2a1*, *Sult2a2*, *Uxs1*, *RT1-Db1*, *Cyp4a3*, *Pstpip1*, and *Il18r1* in certain conditions. Generally speaking, it is reasonably supposed that the consistent genes may be more likely involved in T2D, but we cannot exclude the possibilities of other genes also being involved.

There have been multiple reports concerning the implications of microRNAs in diabetes, but almost all of them focused on the expression profiling, which are mainly related to the intermediate process of disease development [38], [43]–[46]. Here, for the first time we found evidence that microRNAs might be related to T2D by means of copy number variation. We proposed that the altered copy number of *mir-30b* and *mir-30d* in GK rats could contribute to the pathogenesis of T2D. It might occur at the stage of disease initiation: compared with normal Wistar rat, varied copy number of *mir-30b* and *mir-30d* in GK might result in altered expression level at some specific developmental stages and at some specific tissues, and the altered expression of *mir-30b* and *mir-30d* might then lead to dysfunction of some specific targets, contributing to the development of T2D. All these predisposing factors might act in combination as they are involved in this complex disease. In addition to the aforementioned study of the expression of *mir-30d*, there were several other expression profiling reports suggesting the involvement of mir-30 family in diabetes or adipogenesis [47]–[49]. We re-analyzed a public microRNA expression dataset GSE13920, currently the only one microRNA profiling in GK and Wistar rats [43], and found that the expression levels of *mir-30b/30d* in muscle cells were strikingly different between normal rat and T2D rat (Figure S2). The expression change was, however, contradictory to the direction of copy number variation detected here. We noticed that there was a protein-coding gene named *Zfat* which is located at the same gain CNVR as *mir-30b* and *mir-30d* are positioned in. By inspecting the dataset of GSE13271, we found that *Zfat* was up-regulated in liver, but down-regulated in adipose tissues and muscles (Table S13). The down-regulation of *Zfat* in muscles is consistent with that of *mir-30b* and *mir-30d*, that is, all of them are inconsistent with the CNV gain, suggesting further investigations are still needed to confirm these results and to unveil detailed mechanisms.

Although we aimed to find diabetes-specific variants, we noticed that the GK/Wistar CNVRs identified in this work shared quite a few global characteristics with previously reported CNV investigations [19], [21], [30]. For example, our CNVRs from one GK sample accounted for about 1% of the whole genome, non-randomly distributed on the chromosomes, and enriched with genes concerning olfactory transduction and immune response (Table S6), suggesting possible common factors involved in the genesis or maintenance of CNVs. The preference of the GK/Wistar CNVRs for intergenic regions was in accordance with several published reports [19], [50], while some others declared the opposite [21], [51]. It might be due to the distinctiveness of different study subjects or other reasons not revealed currently.

We attempted to explore the causes of T2D of GK rats from CNV data in this study. Admittedly, there were still several aspects not covered here. T2D is a heterogeneous disease, and it may be caused by multiple factors including genetic variations (point variations, structural variations like inversion, translocation, small indels and CNVs) and environmental effects. It is also reported that epigenetic factors may be implicated in the T2D of GK rats [12]. In the present work, only CNVs are concerned, and future studies concerning all these points and validation of the candidates are thus highly anticipated. Array probe design with higher density, hybridizations with more GK individuals, and rat genome annotation with better accuracy will improve the quality of the CNVR data and subsequent analyses. It will be even more promising if next-generation sequencing technology is adopted for CNV discovery, since it can not only define the CNV boundaries more accurately, but also detect inversions or translocations that cannot otherwise be detected by array CGH. In addition, sequencing can identify novel sequence stretches that are not presented in the current reference genome assembly, paving a broader way to identifying T2D and other disease genes.

## Materials and Methods

### Sample Preparation, Array Hybridization and Data Extraction

Three male Goto-Kakizaki rats and 8 male Wistar rats were obtained from SLAC Co., Ltd (Shanghai, China). The rats were anesthetized by formalin at the age of 8 weeks, and the blood was taken from the pericardia and anticoagulated. Genomic DNA was then isolated using DNeasy Blood & Tissue Kit (Qiagen, p/n 69504). All animal experiments were approved by the Biomedical Research Ethics Committee of Shanghai Institutes for Biological Sciences, Chinese Academy of Sciences (IRB00005813). DNA from 3 individuals of GK rat was used as test sample separately, while DNA pooled from 8 Wistar rats served as a common reference.

We used rat genome CGH 244A (Agilent, p/n G4435A) as our oligo aCGH platform, which comprises about 240 K 60-mer probes tiled over the rat genome with the median probe spacing of 7.9 Kb (4.7 Kb in gene regions). Labeling of genomic DNA and hybridization to tiling arrays were performed according to standard Agilent protocols. In brief, 2 µg of genomic DNA from test sample (GK) and 2 µg from reference (Wistar) were digested by AluI/RsaI and labeled by random primer, incorporating Cy5 (red) and Cy3 (green) fluorescent dyes. Test and reference were co-hybridized to the Agilent 244A microarray in the hybridization chamber (Agilent, p/n G2545A) at 65 degrees Centigrade and 20 rpm for 40 hours. For each sample, a dye-swap labeling and hybridization was carried out. In a “forward” hybridization, test and reference samples were dyed with Cy5 and Cy3 respectively, while in the corresponding “reverse” hybridization, test and reference were dyed with Cy3 and Cy5 respectively.

Following hybridization and wash, arrays were imaged using the US80803205 high-resolution scanner (Agilent, p/n G2505B). Fluorescence intensities were extracted using Agilent's Feature Extraction software and used for follow-up processing procedures.

### Data Processing

**Figure 3:**
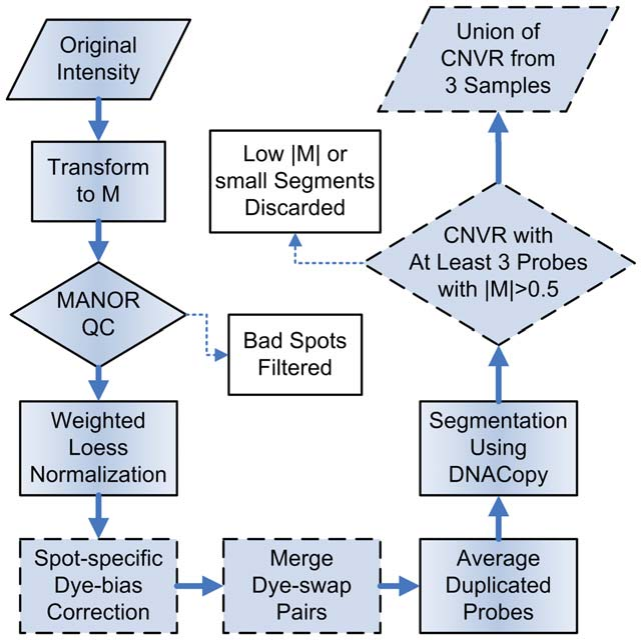
The pipeline of microarray data processing. The shapes bordered by dash-line represent the steps specifically implemented for this study.

The data were processed in the R programming environment (2.9.0), and the snapCGH (1.12.0), limma (1.18.0), MANOR (1.16.0) and DNAcopy (1.18.0) packages from bioconductor project (http://www.bioconductor.org/), and several in-house scripts were adopted for quality control, normalization and segmentation. The data processing framework was mainly based on snapCGH [52], and some necessary modifications were made to fit the requirements of quality control and the dye-swap design. The whole pipeline involving data processing and CNVR identification is illustrated in Figure 3. The signal intensities from the two channels (Cy5 and Cy3) were transformed to the form of logarithm ratios (M values), and were then input to MANOR for quality assessment, especially for adjusting potential global gradient and detecting chip regions with low quality [53]. After that, all M values were normalized with the “loess” coefficients calculated from the M values between −1 and 1 (“weighted loess”) [54], and those from “reverse” hybridizations were multiplied by −1 to make them comparable with those from “forward” ones (*i.e.*, M values of Wistar *vs* GK converted to M values of GK *vs* Wistar).

We further developed a linear model using limma to correct the spot-specific dye bias [55]. For each feature spot, we had 6 M values corresponding to 6 arrays. In our model, each measured M value was written as:
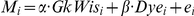
where *i* took values from 1 to 6, representing different arrays. The item of *α* was used to model the contribution of genomic difference between GK and Wistar rats, while the item of *β* was for dye bias. The item of *e* represented the contribution of other factors including difference between GK individuals and random errors. In all hybridizations (*i* = 1, …, 6), *GkWis* took the value of 1. The *Dye* took the value of 1 for “forward” hybridizations (i = 1, 2, 3) and −1 for “reverse” ones (*i* = 4, 5, 6), indicating the different direction of dye bias in “forward” and “reverse” hybridizations. The dye bias correction was to subtract the component of *β·Dye_i_* from M values. By minimizing the sum of square of *e*, we were able to obtain the estimates of *α* and *β* in limma. The dendrograms used to illustrate the necessity of spot-specific dye bias correction were generated by hierarchical clustering with Ward's minimum variance method using Euclidean distances.

After correction of the dye bias, 6 arrays were merged into 3 independent datasets corresponding to 3 GK samples. M values from probe replicates were also averaged. And then segmentation was carried out using DNAcopy package, which aims to fragment the chromosomes into intervals according to the smoothed M values of probes tiled alongside the genome, *i.e.*, the copy number status of the corresponding genome regions [56].

### CNVR Identification

In this work, a CNV region (CNVR) was defined based on the smoothed M values: three or more consecutive probes whose M values were all greater than 0.5 (“gain”) or all less than −0.5 (“loss”) delineated a core region, which extended additional 5 Kb at both sides to define a CNVR. When comparing two diploids, the M value of 0.5 suggests that one of the two alleles is duplicated (log_2_(3/2)≈0.58). That was why 0.5 was set as the M cutoff. The extension of CNVRs for 5 Kb at both sides was due to that 5 Kb is similar to half of the median spacing between consecutive probes on Agilent 244A platform, approximately representing the coverage of a probe at one single side.

When merging CNVRs of multiple samples to a non-redundant set, the criterion was that the region which was detected by at least 1 sample was supposed to be kept in the final set, and overlapped regions be merged, similar to “union” in set operations. Although “union” of 3 samples inevitably resulted in higher false positive rate of CNVR identification, we still preferred high coverage to high precision. The genomic coordinates of CNVRs were referred to the UCSC rn4 assembly (based on RGSC 3.4), and their chromosomal distribution was plotted using Caryoscope [57].

### Investigation into CNVRs

The analyses were also conducted in the R programming environment. The chromosomal locations of NCBI Entrez genes were obtained from the package org.Rn.eg.db (2.2.11) in Bioconductor. Coordinates of genomic features adopted in this work were all referred to the UCSC rn4 assembly, in consistence with those of CNVRs. The CNV genes were identified through genomic interval overlapping, *i.e.*, if one overlapped any of the GK/Wistar CNVRs, it was counted in. In the calculation of the length of gene regions in the CNVRs or in the whole genome, overlapping gene regions were merged to non-redundant intervals to avoid duplicated counting.

The random simulation for estimating the statistical significance of CNVRs' preference to intergenic regions was designed as follows. The same counts of genomic intervals as CNVRs (137 in this study) were randomly chosen from the rat chromosomes, and their lengths were also the same as the corresponding CNVRs. The summed length of the overlaps between them and Entrez gene regions was then calculated as the statistic. After 1000 rounds of this process, an empirical distribution representing the overlap magnitudes between gene regions and random genomic intervals was obtained. And thus the probability of the overlap length less than that between real CNVRs and gene regions (3.22 Mb in this study) could be estimated.

The T2D-related gene list was compiled from an article which collected 172 human T2D-related genes [10] and the T2D-DB database [9] which contained 330, 60, and 36 genes from human, mouse and rat, respectively. Using NCBI HomoloGene Release 64 (http://www.ncbi.nlm.nih.gov/homologene), we obtained the corresponding rat homologues of human and mouse genes. A total of 425 non-redundant rat genes turned out to form the T2D-related gene list.

In addition to org.Rn.eg.db (2.2.11), the bioconductor packages including KEGG.db (2.2.11), and GOstats (2.10.0) were used to obtain the gene to pathway mappings, and to test the significance of pathway enrichments. The “near-T2D” gene set was prepared from a study on human “diseasome” [31], where all the diseases were organized in a inter-connected network. We collected all the diseases that directly connected to T2D in this network, and then all the genes corresponding to these diseases were retrieved as the “near-T2D” gene set. The rat homologues of “near-T2D” human genes were also obtained from the NCBI HomoloGene release 64, resulting in 1097 rat “near-T2D” genes. The rat QTL data were from the source of RGD [58], and their overlap with CNV genes were checked through navigating the UCSC genome browser manually [59]. Tiffin's human T2D gene set [33] were originally represented by Ensembl gene IDs and were converted to Entrez gene IDs using org.Hs.eg.db (2.2.11). The human homologues of the rat CNV genes were obtained using HomoloGene release 64 as well, which made feasible the comparison between CNV genes and Tiffin's dataset.

The locations of ultraconserved elements on rat genome (assembly rn4) were determined using BLAT [60]. The 481 published elements resulted in 484 positions due to non-unique mapping. The simulation for estimating the significance of the overlap between CNVRs and the ultraconserved elements was conducted following the previous procedure for overlap between CNVRs and gene regions.

Rat microRNAs along with their genomic coordinates based on assembly rn4 were downloaded from miRBase (http://www.mirbase.org/, release 14) [61]. Predicted targets of mature microRNAs were obtained from MicroCosm (http://www.ebi.ac.uk/enright-srv/microcosm/htdocs/targets/v5/) based on the miRanda algorithm [39]. Since *rno-mir-30b* was processed to two mature forms, *rno-miR-30b-3p* and *rno-miR-30b-5p*, their targets were combined for further analysis; and it was the same with *rno-mir-30d*, where the targets of *rno-miR-30d* and rno-miR-30d* were merged. The targets in MicroCosm presented as Ensembl transcript IDs were converted to Entrez gene IDs utilizing the mappings provided by the package org.Rn.eg.db. The significance of the targets' enrichment in the 425 T2D-related genes was calculated in a hypergeometric distribution by taking all the genes in the package “org.Rn.eg.db” as background. The pathway mapping and enrichment analysis of targets were performed as the same as those of CNV genes using the package of GOstats.

The super series GSE13271 contains three expression datasets: GSE13268 (adipose), GSE13269 (muscle), and GSE13270 (liver), all of which profiled the gene expressions of GK and Wistar rats in normal and high fat diet, and in 5 time points, thus resulting in 30 conditions. For genes with more than one probeset, we kept the one which was most often associated with the highest expression level. If there were still more than one probeset left, the intensities of the remaining probesets were averaged to represent the expression level of this gene. We then performed *t*-test to identify differentially expressed genes between GK and Wistar samples, and calculated the base 2 logarithm of the fold changes for the expression levels. FDR correction using BH method [62] was adopted to adjust the raw *p*-values of multiple hypothesis testings, and 0.2 was chosen as the threshold, which means that more than 80% of the identified genes are truly differentially expressed. As for the microRNA expression dataset GSE13920, we simply looked at the mean signal intensities after removing mean background noise for each probe of *mir-30b* and *mir-30d*.

## Supporting Information

Figure S1 Effect of dye bias correction. The samples were clustered according to the corresponding M values, before (A) and after (B) correcting the systematic errors caused by dye bias, respectively. GK1′, GK2′ and GK4′ represent the corresponding “reverse” hybridizations for GK1, GK2, and GK4, respectively.
(0.08 MB TIF)

Figure S2 Expression levels of mir-30b/30d in the muscle of GK and Wistar Rat. Data were downloaded from GEO (GSE13920), and two GK samples and 2 Wistar samples were hybridized on 4 single-channel microarrays respectively. The expression level was represented by the mean foreground signal intensity after subtracting the mean background signal intensity. Each probe duplicated 3 times.
(0.22 MB TIF)

Table S1 CNVRs identified in 3 GK rat DNA samples.
(0.05 MB XLS)

Table S2 Simple statistics of GK/Wistar CNVRs in 3 samples.
(0.04 MB DOC)

Table S3 Common CNVRs between samples (Mb).
(0.03 MB DOC)

Table S4 Non-redundant CNVRs and Entrez genes directly overlapped them.
(0.06 MB XLS)

Table S5 Genes identified from GK/Wistar CNVRs.
(0.03 MB XLS)

Table S6 The pathway mapping and enrichment of CNV genes.
(0.03 MB XLS)

Table S7 CNV genes occurred in “near-T2D” diseases.
(0.02 MB XLS)

Table S8 Preferentially selected protein-coding genes in GK/Wistar CNVRs.
(0.02 MB XLS)

Table S9 Logarithm (base 2) ratios of CNVRs (GK vs. Wistar) containing microRNAs.
(0.01 MB XLS)

Table S10 Pathway mapping and enrichment of the targets of rno-mir-30b.
(0.04 MB XLS)

Table S11 Pathway mapping and enrichment of the targets of rno-mir-30d.
(0.04 MB XLS)

Table S12 Coding signals annotated in sources other than Entrez Gene (EG).
(0.03 MB DOC)

Table S13 Comparison of CNV genes and differentially expressed genes.
(0.06 MB XLS)
