# Supplementary figures and images for: Analyses of Copy Number Variation of GK Rat Reveal New Putative Type 2 Diabetes Susceptibility Loci
Source: PLoS One. 2010 Nov 23;5(11):e14077. doi: 10.1371/journal.pone.0014077 (PMC2990713; doi:10.1371/journal.pone.0014077)

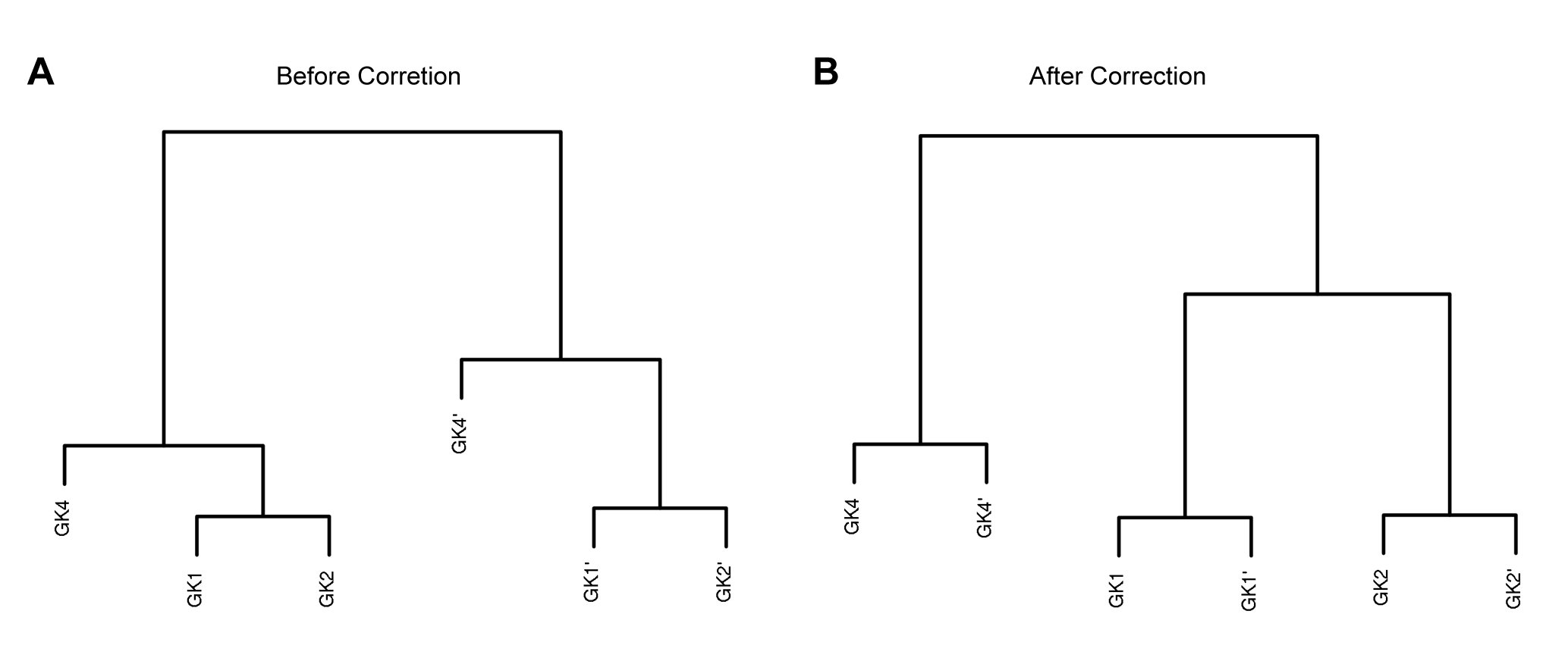

Supplement: Figure S1 — Effect of dye bias correction. The samples were clustered according to the corresponding M values, before (A) and after (B) correcting the systematic errors caused by dye bias, respectively. GK1′, GK2′ and GK4′ represent the corresponding “reverse” hybridizations for GK1, GK2, and GK4, respectively. (0.08 MB TIF) [file pone.0014077.s001.tif]

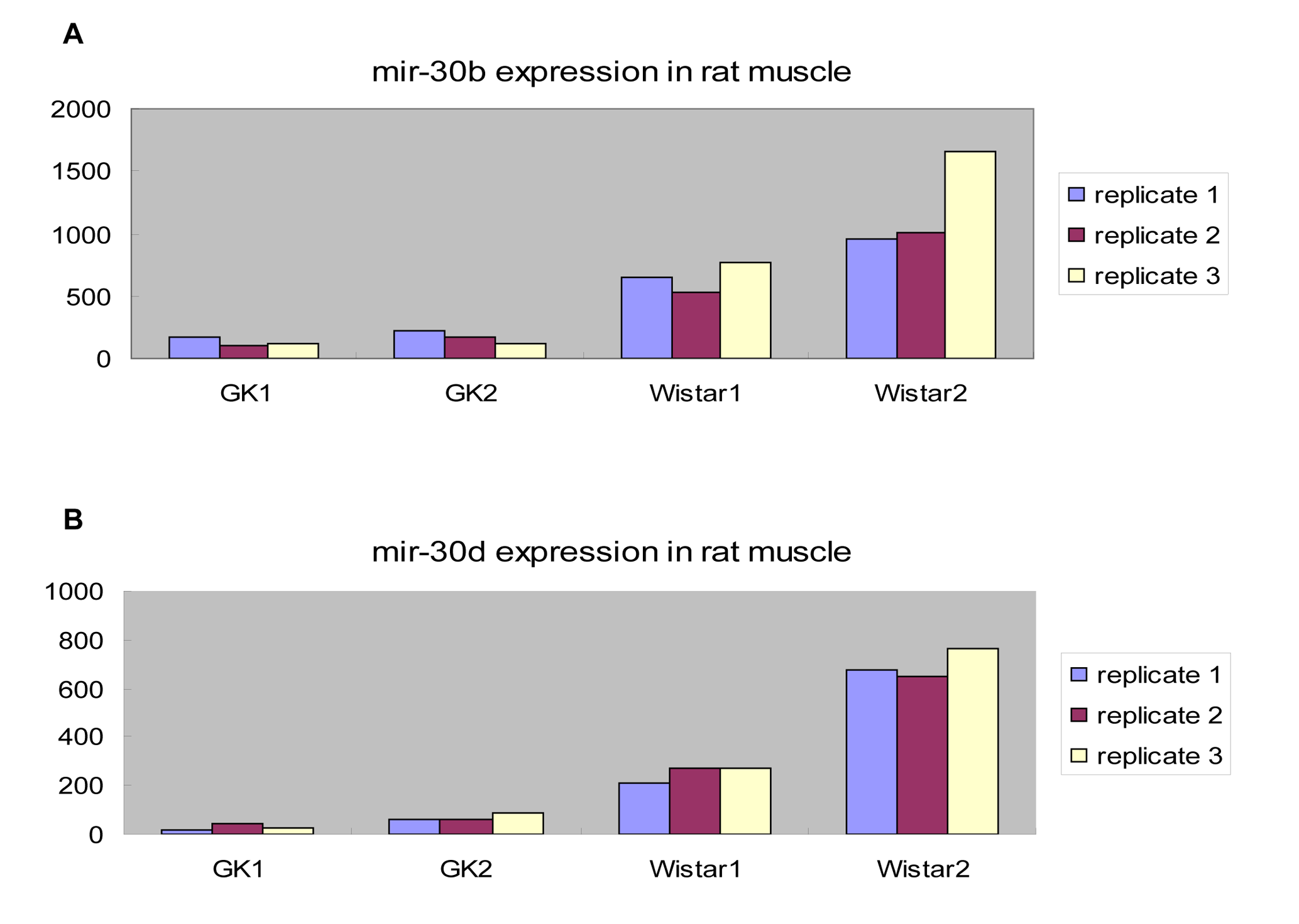

Supplement: Figure S2 — Expression levels of mir-30b/30d in the muscle of GK and Wistar Rat. Data were downloaded from GEO (GSE13920), and two GK samples and 2 Wistar samples were hybridized on 4 single-channel microarrays respectively. The expression level was represented by the mean foreground signal intensity after subtracting the mean background signal intensity. Each probe duplicated 3 times. (0.22 MB TIF) [file pone.0014077.s002.tif]
