# Supplementary material for: Analyses of Copy Number Variation of GK Rat Reveal New Putative Type 2 Diabetes Susceptibility Loci
Source: PLoS One. 2010 Nov 23;5(11):e14077. doi: 10.1371/journal.pone.0014077 (PMC2990713; doi:10.1371/journal.pone.0014077)
Supplement: Table S2 — Simple statistics of GK/Wistar CNVRs in 3 samples. (0.04 MB DOC) [file pone.0014077.s004.doc]

Table S2. Simple statistics of GK/Wistar CNVRs in 3 samples ^a^

|  | GK1 | GK2 | GK4 | Average | Non-redundant ^b^ |
| --- | --- | --- | --- | --- | --- |
| Total Length (Mb) | 27.48 | 28.84 | 23.16 | 26.49 | 36.31 |
| Total CNVR counts | 104 | 103 | 96 | 101.0 | 137 |
| Median Size (Kb) | 84.93 | 74.75 | 73.88 | 77.85 | 67.81 |
| (+) Length (Mb) | 16.95 | 18.89 | 11.03 | 15.62 | 22.75 |
| (+) CNVR counts | 60 | 59 | 44 | 54.33 | 73 |
| (+) Median Size (Kb) | 113.97 | 89.09 | 73.88 | 93.65 | 91.57 |
| (-) Length (Mb) | 10.53 | 9.95 | 12.13 | 10.87 | 13.56 |
| (-) CNVR counts | 44 | 44 | 52 | 46.67 | 64 |
| (-) Median Size (Kb) | 53.29 | 47.50 | 66.95 | 55.91 | 47.46 |

^a^ Three samples were labeled with "GK1", "GK2" and "GK4".

^b^ The non-redundant set of CNVRs by merging overlapping CNVRs from different samples, similar with the "union" in set operation.

'+': gain; '-': loss.
