# Supplementary material for: Analyses of Copy Number Variation of GK Rat Reveal New Putative Type 2 Diabetes Susceptibility Loci
Source: PLoS One. 2010 Nov 23;5(11):e14077. doi: 10.1371/journal.pone.0014077 (PMC2990713; doi:10.1371/journal.pone.0014077)
Supplement: Table S3 — Common CNVRs between samples (Mb). (0.03 MB DOC) [file pone.0014077.s005.doc]

Table S3. Common CNVRs between samples (Mb)

|  | GK1 | GK2 | GK4 |
| --- | --- | --- | --- |
| GK1 | 27.48 | - | - |
| GK2 | 23.48 | 28.84 | - |
| GK4 | 19.26 | 18.30 | 23.16 |
