# Supplementary material for: Analyses of Copy Number Variation of GK Rat Reveal New Putative Type 2 Diabetes Susceptibility Loci
Source: PLoS One. 2010 Nov 23;5(11):e14077. doi: 10.1371/journal.pone.0014077 (PMC2990713; doi:10.1371/journal.pone.0014077)
Supplement: Table S12 — Coding signals annotated in sources other than Entrez Gene (EG). (0.03 MB DOC) [file pone.0014077.s014.doc]

Table S12. Coding signals annotated in sources other than Entrez Gene (EG)

| Annotation source | EG region | Region excluding EG |
| --- | --- | --- |
| KnownGene | 48 | 10 |
| RefGene | 134 | 1 |
| EnsemblGene | 193 | 362 |
| mRNA | 168 | 431 |
| EST | 2547 | 2401 |
